# The Mechanisms of Peripheral Nerve Preconditioning Injury on Promoting Axonal Regeneration

**DOI:** 10.1155/2021/6648004

**Published:** 2021-01-06

**Authors:** Xiaoyan Yang, Ruixuan Liu, Ying Xu, XiangYu Ma, Bing Zhou

**Affiliations:** ^1^Beijing Advanced Innovation Center for Big Data-Based Precision Medicine, Beihang University, Beijing 100191, China; ^2^Interdisciplinary Innovation Institute of Medicine and Engineering Interdisciplinary, Beihang University, Beijing 100191, China

## Abstract

Two major factors contribute to the failure of axonal regrowth in the central nervous system (CNS), namely, the neuronal intrinsic regenerative capacity and the extrinsic local inhibitory microenvironments. However, a preconditioning peripheral nerve lesion could substantially enhance the regeneration of central axons following a subsequent spinal cord injury. In the present review, we summarize the molecular mechanisms of the preconditioning injury effect on promoting axonal regeneration. The injury signal transduction resulting from preconditioning peripheral nerve injury regulates the RAG expression to enhance axonal regeneration. Importantly, preconditioning peripheral nerve injury triggers interactions between neurons and nonneuronal cells to amplify and maintain their effects. Additionally, the preconditioning injury impacts mitochondria, protein, and lipid synthesis. All these coordinated changes endow axonal regeneration.

## 1. Introduction

The axons of neurons in the peripheral nervous system (PNS) retain considerable regenerative capacity following injury, while the axons of neurons in the adult central nervous system (CNS) fail to regrow spontaneously [1]. Two major factors contribute to the failure of axonal regrowth in the CNS: neurons losing their intrinsic regenerative capacity after maturation, and the inhibition of local microenvironments preventing axon growth [2, 3]. Studies have demonstrated that the elimination of external inhibitory molecules is not sufficient for promoting long distance axonal regeneration [4]. Thus, the low intrinsic regenerative capacity with aging is the main obstacle for central axonal regeneration [5–12].

**Figure 1:**
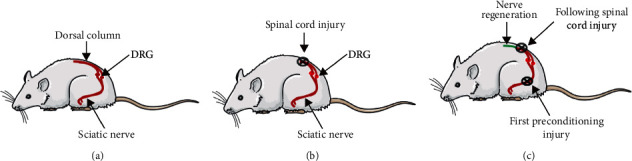
Preconditioning injury paradigm. (a) The DRGs are pseudounipolar neurons, dividing their axons into peripheral and central branches. The peripheral branches aggregate into the sciatic nerve to innervate sensory targets, and the central branches enter into the dorsal column and relay sensory information to the brain stem. (b) The central branch of DRG failed to regenerate after spinal cord injury. (c) A prior injury of the sciatic nerve could enhance the regrowth capacity of DRG to elicit regeneration of axons in the central nerve system following a subsequent spinal injury.

The primary sensory neurons are pseudounipolar neurons that extend their axons into peripheral and central branches. The peripheral branches project to innervate sensory targets, and the central axonal branches enter into the dorsal column and relay sensory information back to the brain stem. Thus, the primary sensory neurons, with their cell bodies in the dorsal root ganglia, are ideal for investigating regeneration mechanisms following axotomy both in vivo and in vitro [13]. Interestingly, a prior injury of peripheral branches permits regeneration of axons in CNS following a subsequent injury, despite the existence of an inhibitory environment in the CNS, through the activation of intrinsic regeneration capacity, which is referred to as the preconditioning injury effect [14–17](Figure 1). The transection of DRG axons in the central nerve systems did not elicit the preconditioning injury effect similar to the peripheral axons axotomy [13].

The initial findings have driven the speculation that the distinct regenerating response is due to intrinsic differences in postinjury changes between the peripheral and central nerve axotomy [16], leading to significant concern and propelled molecular mechanism studies focused on the multiple genetic regulators of the axon regeneration capacity. Accordingly, many regeneration-associated genes (RAG) have been certified, such as ATF3 (activating transcription factor 3), Sprr1a, c-Jun, and Smad1 [18–20]. However, individual or combined axonal RAG regulation failed to induce the full axon regeneration in CNS, suggesting a permissive role of RAGs [18].

Recently, with technological advances in bioinformatics and high-throughput gene sequencing, an increasing body of evidence shows that epigenetic regulation for DNA accessibility and the transcriptional program are synergistic with PNS injury, while they are less affected by CNS injury [20, 21]. In addition to RAG dynamics, a significant concern of the damage response may also be related to the interaction between neurons and nonneuronal cells after axon axotomy [22]. Interestingly, the metabolic pathways and mitochondrial behavior regulation may also contribute to preconditioning injury effects, indicating enhanced cellular metabolism adaptation during axon regeneration, which caught extensive attention [23, 24]. Understanding the molecular mechanisms of preconditioning injury may ultimately benefit novel intervention to improve CNS recovery after injury. In the present review, we provide an overview of molecular and cellular mechanisms on preconditioning injury, including injury signal transduction, epigenetics modification, neuroinflammation, and immune response.

## 2. Injury Signal Transduction of the Preconditioning Injury Effect

With the preconditioning peripheral nerve injury induction, the adult DRG's axon growth capacity is revived, and the neurons are reprogramming into a proregenerative state, both in vivo and in vitro. Over the past decades, comprehensive studies indicate that peripheral nerve injury signals can retrograde back to the DRG cell bodies and initiate the neuronal genetic program responsible for enhancing axon growth. Injury signal transduction is encoded by rapid changes in Ca^2+^ fluxes in the injured neuron and some other slower signals that are conveyed by axoplasmic transport [25].

**Figure 2:**
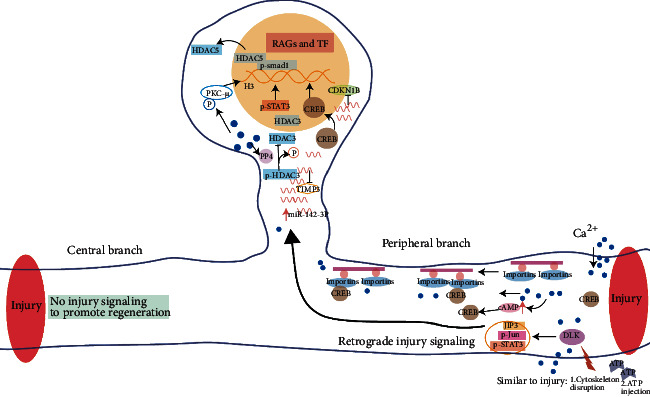
Signal transduction of peripheral nerve preconditioning injury regulates the RAG expression. Retrograded injury signaling after sciatic nerve injury includes calcium wave, a sensor of axon injury DLK, and axoplasmic importins. The injury signaling elicit genetic program to express RAGs and TFs responsible for enhancing axon growth. RAGs: regeneration-associated genes; TFs: transcription factors; CDKN1B: cyclin-dependent kinase inhibitor 1B; TIMP3: tissue inhibitor of metalloproteinase 3.

Studies of preconditioning injury have provided evidence that Ca^2+^ availability may regulate histone acetylation to enhance axon regrowth via HDAC5 and HDAC3. In the context of sciatic nerve injury, the peripheral axonal membrane breaks, and then ionic calcium flows into the cell. A back-propagating calcium wave to soma stimulates histone deacetylase 5 (HDAC5) export from the nucleus via protein kinase C-*μ* activation, which in turn boosts histone acetylation to reprogram the chromatin for subsequent transcription events [26]. As injury-regulated tubulin deacetylase, HDAC5 also plays an essential role in tubulin deacetylation at the injury site to regulate growth cone dynamics and axon regeneration [27]. Unlike PNS injury, the HDAC5 pathway cannot be activated in a model of CNS injury. The calcium increase induced by peripheral targeted nerve injury activates protein phosphatase 4 (PP4) to dephosphorylates HDAC3, resulting in inhibiting the HDAC3 activity and therefor promoting axonal regeneration through enhanced histone acetylation. PP4-dependent HDAC3 dephosphorylation is pivotal to regenerative success [28](Figure 2).

The cAMP was one of the major downstream effectors of calcium during axonal injury. Studies have shown that a preconditioning peripheral nerve injury activates the cAMP signaling pathway to improve growth capacity to overcome inhibition by myelin-associated glycoprotein (MAG) and myelin [29]. Endogenous cAMP levels in rat DRG are related to regenerative capacity [30]. The neurite outgrowth of young neurons is dramatically reduced by inhibiting protein kinase A (PKA, a downstream effector of cAMP), and increasing cAMP overcomes myelin-MAG inhibition for older neurons. Jin Qiu et al. [29] found that 1 day after peripheral nerve injury, cAMP levels were increased in DRG to overcome MAG/myelin's inhibitory effect on nerve regeneration dependent on PKA. Injection of db-cAMP can simulate a preconditioning injury effect. Elevated cAMP activated cAMP-responsive element-binding protein 1 (CREB1) to upregulate Arg I, leading to an increasing in polyamine synthesis [31].

A sensor of axon injury DLK (MAP3K, dual leucine zipper kinase), which is independent of calcium concentration changes, is responsible for the retrograde injury signal [32]. Lack of DLK reduces the upregulation of activated transcription factors such as phosphorylated JUN and STAT3 in sensory neuron cell bodies because DLK is required for retrograde transport of p-STAT3 at the site of axon injury to the cell body [32]. DLK and JNK are linked to the axon transport machinery by a scaffolding protein JIP3. Retrograde transport of JIP3 was also shown to be perturbed in DLK-knockout axons. A study has demonstrated that DLK is required for JNK-dependent retrograde injury signaling and also shows that it regulates other retrograde cargoes [32]. A recent study suggests that HSP90 is required for DLK functions in proregenerative axon injury signaling. As a chaperone protein, HSP90 binds DLK to inhibit rapid DLK protein degradation and stable it in the sciatic nerve [33]. Other groups had demonstrated that after sciatic nerve injury, STAT3 is phosphorylated rapidly and retrogradely transported to the nucleus and initiate transcription of target genes [34, 35], while STAT3 retrograde transport was attenuated in DLK-knockout axons [32]. Using a DLK-knockout mouse model that underwent DRG conditional injury, Shin's lab [36] also indicated that the gene expression changes on the DLK pathway according to the time course through gene ontology analysis. Su-Hyuk Ko and his colleague found DLK-1-mediated injury-triggered autophagy activation to promote axon regeneration [37].

Axoplasmic importins are DLK-independent signaling molecules sensing retrograde injury signaling in the injury nerve [38]. The importin *β*1 protein was presented and increased as a result of local translation of axonal mRNA after injury. This subsequently leads to the generation of high-affinity a NLS-binding complex that is transported retrogradely with the moto protein dynein to modulate the axonal regeneration (Figure 2). Furthermore, a study showed that axon-derived Luman/CREB3 also played an important role in transducing retrograde regeneration signal after axonal injury. Notably, CREB3 synthesis and releasing from the axonal endoplasmic reticulum was induced by axotomy and then transferred to the cell nucleus in an importin-mediated manner [39], which regulates the unfolded protein response (UPR^ER^) and cholesterol biosynthesis that are crucially associating with the acute stress response in axonal growth [39, 40]. The authors also show that upregulation and nuclear localization of Luman coordinate with the increased transcriptional activity in injured neurons, achieving maximal outgrowth capacity at two-day injury-conditioned neurons relative to naïve [41].

In addition to sciatic nerve transection, Vera Valakh and colleges found that the perturbation of actin or cytoskeleton damaged by pharmacological agents also activated the DLK pathway (Figure 2). This activation of the DLK pathway may enhance axon regeneration akin to preconditioning [42]. Since the preconditioning injury can cause ATP release from axons and Schwann cells, some postulation suggested that ATP could also be the key injury signal to transmit injured neurons into a regeneration state [43]. One study shows that ATP injection or precondition injury increased the expression of phospho-STAT3 and GAP43, indicating that P2Y2 receptors are involved in the activation of STAT3 [44].In another study, through applying a cAMP agonist rolipram, the author elucidates that the mechanisms of low-frequency electrical stimulation are to upregulate neurotrophic factors and cAMP to accelerate nerve regeneration [45].

## 3. Preconditioning Injury Regulates the RAG Expression to Enhance Axon Regeneration

In contrast to transient signaling evoked by injury, epigenetic regulation, which refers to gene expression changes without altering underlying DNA sequences, is involved in the transcriptional profiling of the preconditioning injury effect [46]. Finelli MJ and coworkers established a correlation between histone acetylation and intrinsic axon growth capacity in adult DRG neurons [47]. They identified a transcriptional complex consisting of pSmad1 and other histone-modifying enzymes, which involved in the restoration of a subset of early RAG promoter histone acetylation and expression induction [47]. Consistently, when neurons are transmitted into a growth state with H4 acetylation, a set of target genes of Smad1 is restored in the preconditioning injury paradigm. Interestingly, Smad1, a conserved transcription factor (TF) downstream of bone morphogenetic protein (BMP) signaling, activated by peripheral nerve axotomy in adult sensory neurons, is correlated with neurotrophin-mediated axon regeneration in vitro and in vivo [48], while the central axotomy procedure of DRGs fails to activate the Smad1 pathway [49].

Histone acetylation and chromatin accessibility characterize injury discrepancy after PNS or CNS axonal injury. Specifically, the H3K9ac, H3K27ac, and H3K27me3 were modified differently in response to peripheral nerve axotomy and CNS axotomy. These modifications correlate with the various regenerative abilities of sensory neurons [21]. As mentioned above, the retrograde propagation of calcium wave from axotomy in DRG neurons elicits nuclear export of HDAC5, leading to elevated H3 acetylation and RAGs induction [26]. Interestingly, the expression of HIF1*α* in DRG neurons is necessary and sufficient for histone H3 acetylation to promote peripheral axon regeneration in a sciatic nerve injury model [50]. Moreover, systematic epigenetic studies showed that the histone acetyltransferase p300/CBP-associated factor (PCAF) promotes acetylation of histone 3 Lys 9 at the RAG promoters following a peripheral but not a central axonal injury [51]. Additionally, PCAF phosphorylation was required for retrograde transport of extracellular signal-regulated kinase (ERK) and nuclear localization after peripheral nerve axotomy, which provides a connection of the transduction pathway of injury signals from the site of axotomy to nuclear and chromatin modifications [51]. A recent study suggested that Creb-binding protein- (Cbp-) mediated histone acetylation increased the expression of RAGs [52]. Induction of Tet3 and elevated 5hmC levels in adult DRG after peripheral nerve axotomy is also correlated to induction of RAGs such as ATF3, Smad1, and STAT3 [53, 54].

Besides epigenetic alterations, various RNAs regulating translation after transcription have been found to play an important role in nerve regeneration. With the development of RNA sequencing, microRNAs (miRNAs), a novel class of small noncoding RNAs, were found to regulate posttranscription of the expression. Wu et al. identified the upregulation of miR-142-3p following sciatic nerve injury in rat DRG. miR-142-3p binds the 3′-UTR of cyclin-dependent kinase inhibitor 1B (CDKN1B, also as p27Kip1) and tissue inhibitor of metalloproteinase 3 (TIMP3), to regulate their expression for appropriate nerve regeneration [55]. The long noncoding RNA (LncRNAs) expression also changes markedly after nerve injury [56]. Circular RNAs (circRNAs), single-stranded regulatory RNAs participating in regulating transcription and splicing, were increased after sciatic nerve injury in rat via a quantitative real-time polymerase chain reaction. circ-Spidr, a kind of circular RNAs, partially modulated the PI3K-Akt pathway to control DRG axon regeneration in vitro and in vivo [57].

With the employing of system biology and bio-information approaches, Chandran et al. identified core networks that specifically change after PNS versus CNS injury. The major upregulated TFs after PNS injury include ATF3, EGR1, FOS, JUN, MYC, RELA, SMAD1, and STAT3. ATF3 and JUN are the top two hub TFs present in the core regeneration-associated gene network [20]. Another study suggested that peripheral axonal injury activated ATF3 to increase DRG neurite elongation in vitro. However, with the ATF3 transgenic mice, Seijffers et al. showed that ATF3 contributes to the intrinsic growth of injured neurons, while these neurons fail to overcome the environmental inhibitory effects [19].

Similarly, transcriptomic analysis suggested that sciatic nerve injury triggers the mRNA level change in the spinal cord [58]. Some of these regulated mRNAs are involved in cell growth and development. Combining axoplasmic proteomics with cell body RNA-seq, Guiping Kong and colleagues found that AMPK*α*1 is specifically downregulated and contributes to enhanced axonal regeneration following sciatic injury, different from injured central projecting of DRG [59].

## 4. Interactions between Neurons and Nonneurons from Preconditioning Injury

In consistent with the observed inflammation and immune response from the bioinformatic analysis [60], neuroimmunology regulation and the interaction between neurons and nonneurons were involved in the PNS preconditioning effect [22]. Inflammation-derived signals could also have an impaction on the intrinsic growth ability of DRG.

**Figure 3:**
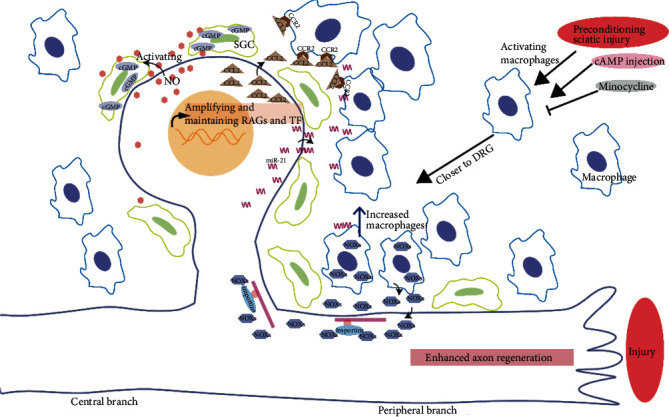
Interaction between neurons and nonneurons after PNS preconditioning injury. The preconditioning sciatic nerve injury increased the number of macrophages and inflammatory mediators and drives macrophage activation toward DRG. The interactions amplify and maintain RAGs and TF expression to enhance axon regeneration. SGC: satellite glial cells; CCL2:C-C motif chemokine ligand 2; CCR2: C-C motif chemokine receptor 2; NOXs: NADPH oxidase 2 complexes.

As a highly dynamic pathogenic process, the number of macrophages in DRGs and inflammatory mediators increased after sciatic nerve transection in rats. The preconditioning sciatic nerve injury may trigger neuron–macrophage interactions in the DRGs to drive macrophage activation toward a proregenerative phenotype [61], and macrophages tend to be closer proximity to small and large neurons in DRG [62] (Figure 3). Intriguing, the upregulation and release of miR-21 from DRG neurons after nerve injury contribute to sensory neuron–macrophage communication [63]. Minocycline, a macrophage inhibitor, could limit the number of macrophages and downregulate inflammatory mediators, coincide with abolished regenerative capacity enhancement by conditioning injury in vitro and in vivo [61]. The BDNF-cAMP pathway-dependent cell-cell interaction pathways have been identified as well. The upregulation of macrophage-derived neurotrophin BDNF is induced by preconditioning sciatic nerve injury [61]. Neutralization of endogenous BDNF impairs the enhanced neurite growth and regeneration of DRG in vitro and in vivo [64], while the injection of cAMP also increases the macrophages. Besides, recent findings with RNA sequencing suggested that, after nerve injury, satellite glial cells (SGC) contain abundant genes involving in the immune system and cell-to-cell communication [62]. However, these genes changed at different time points, leading to the speculating that macrophages may result from the injury-induced proliferation of SGCs [62]. These studies indicated the importance of neuron-macrophage interactions, and that macrophages played a crucial role in maintaining regenerative capacity.

Wang et al. [65] reported that phospho-JUN is also involved in triggering the expression of Sarm1 and several chemokines in DRG neurons, including C-C motif chemokine ligand 2 (CCL2). CCL2 (also known as monocyte chemoattractant protein-1) was highly expressed in DRG 48 hours after nerve injury [66]. Upon preconditioning injury, CCL2 releasing from neurons activates CCR2 of macrophages to mediate neuron–macrophage interactions (Figure 3). Macrophage accumulation around axotomized cell bodies resulting from an injury of the sciatic nerve but not the dorsal root is necessary for a peripheral nerve preconditioning injury effect via a STAT3-dependent mechanism [67], since the CCL2 overexpression led to a selective increase in leukemia-inhibitory factor (LIF) mRNA and neuronal phosphorylated STAT3 (pSTAT3) in L5 DRGs. pSTAT3 is involved in DLK-dependent injury signal transduction [32]. However, how the injury of the sciatic nerve stimulates neurons to release CCL2 is unknown. Sigma-1 receptor (Sig-1R), expressed by DRG neurons, plays an important role in DRG neuron-macrophage/monocyte communication after sciatic nerve injury. Following the chemokine CCL2 produced by DRG neurons after nerve injury, macrophage/monocyte infiltration is mainly located around injured DRG with translocated Sig-1R in WT mice. In contrast, reducing levels of CCL2 and decreasing macrophage/monocyte infiltration were observed in DRG of Sig-1R knockout mice [68]. Cobos et al. [69] reported a number of chemokines increases, including CCL4, CCL7, and CCL9 after injury in the DRG with RNA seq study.

Additionally, preconditioning injury also has an impact on the remote DRG. Dubovy et al. found that 7 days after sciatic nerve injury, the ulnar nerve with subsequent crush regrows longer than with ulnar nerve crush only [70]. They speculated that DRG neurons in the cervical were *trans*-activated into the proregenerative state by preconditioning sciatic nerve injury through the IL-6 signaling pathway [71]. Highlighting the inflammatory cytokines IL-6 has also participated in preconditioning effects. After sciatic nerve injury, the IL-6 mRNA expression correlated with the increased macrophages in a similar time course [61]. Besides, a study shows that macrophages were the primary source of IL-6 by detecting IL-6 only in CD68-positive macrophages [61]. Cao et al. [72] have shown that cytokine interleukin-6 (IL-6) is upregulated in culture DRG neurons after cAMP treatment or a preconditioning injury. And this increase in IL-6 is sufficient to overcome the myelin-associated inhibitors and promote spinal axon regeneration in vivo, mimicking the preconditioning injury, although IL-6 is not an essential component of these responses [72]. IL-6 reactivates the expression of RAGs, such GAP-43, Sprr1a, and Arginase I and triggers the mTOR pathway in neurons surrounding the injury site to promote axonal regrowth [73]. The increased IL-6 is through the classic IL-6 trimeric receptor to activate the JAK signaling cascade. Together, the interaction between neurons and nonneurons mediated by chemokine signaling is necessary and sufficient for mimicking the preconditioning injury, which may coordinate with the retrograde signaling initiated from the injury site, to corroborate the successful axonal regeneration.

In addition to the regulated cytokines, reactive oxygen species (ROS) also play an important role in intraganglionic communications. Nitric oxide (NO), one of the reactive oxygen species, as a messenger released by sensory neurons after axotomy, activates satellite glial cells (SGCs) in DRG and induces cyclic GMP (cGMP) production in these SGCs [74]. Accordingly, NO also reinforced Ca^2+^ wave propagation in DRG cultures. Another study has shown that the sciatic nerve injury triggers NADPH oxidase 2 complexes (NOXs) releasing from macrophages, and then NOXs are endocytosed into injured axons [75]. Endosomal NOXs are transported to the cell body via importins and produced ROS to oxidize PTEN, which inhibited PTEN to promote axonal regeneration and functional recovery after spinal injury.

Other non-neuron cells are also involved in nerve regrowth, such as Schwann cells (SCs) and endothelial cells. The LDL receptor-related protein-1 (LRP1) is the cell-signaling receptor required for normal SC function. Comparing scLRP1-/- mice with wild-type littermates with and without peripheral nerve injury, Poplawski et al. provide evidence that SCs regulate the RAG expression in DRGs [76]. In addition, other cell type endothelial cells are also involved in nerve regrowth. Macrophages may selectively sense hypoxia and induced blood vessels via VEGF-A to alleviate the hypoxia in the process of reconnecting a severed nerve in PNS [77].

These interactions are essential for amplifying and maintaining the RAG expression and enhance regenerative capacity by preconditioning peripheral nerve injury [78]. Maintenance of the RAG expression is crucial for successful nerve regeneration [79].

## 5. The Effects of Preconditioning Injury on Mitochondria, Protein, and Lipid Synthesis

Kiryu-Seo et al. observed that mitochondrial fission increased in injured motor axons after sciatic nerve transection [80]. Simultaneously, the anterograde transport of mitochondrial increased in proximal segments of injured intercostal nerves with a vigorous growth response [81]. A preconditioning peripheral nerve injury also upregulated the levels of molecular motors, polyglutamylated, and tyrosinated tubulin to enhance mitochondria transport in both central and peripheral branches of DRG, which support a rapid and sustained central axon regeneration [82]. However, spinal cord injury cannot elicit increased mitochondrial transport [82]. Suggesting altered mitochondrial transport could be the potential intervention target. Zhou et al.'s previous studies suggest that enhancing mitochondrial transport might rescue energy deficits to promote axon regrowth [83], while the relationship between mitochondrial as a powerhouse and the preconditioning effect during nerve regrowth still needs to be investigated and answered.

In addition to the mitochondria, the preconditioning injury also impacts protein synthesis and lipid metabolism in injured neurons. The priming of peripheral nerve injury, not a central nerve lesion of rat DRGs, increases metabolic enzymes such as NADH dehydrogenase and catalase, which is detected by radiolabeling and mass spectrometry [82]. The preconditioning PNS injury promotes protein synthesis via enhancing the rapamycin-insensitive mTOR activity [84] and m6A signaling in adult DRGs [85]. The level of neuronal diglyceride acyltransferases (DGATs) was deceased during injury to switch from triglycerides to phospholipid synthesis, which facilitates axon regeneration [24]. Similarly, an upregulation of cAMP induced by sciatic transection initiated the expression of arginase 1 (Arg), a rate-limiting enzyme in the synthesis of the polyamines, in a cAMP response element-binding protein (CREB)-dependent manner [31, 86]. Activation of CREB is also required for cAMP to upregulate Arg I that increased polyamine synthesis and improves axonal regeneration on the inhibitory substrate.

## 6. Conclusion

Previous studies have advanced how preconditioning peripheral axon injury elicits widespread regulations, including injury signals transduction, gene expressions, epigenetic modification, and neuroinflammation to facilitate neuron regeneration. Considerable progress has been made in recent years. However, the coordination and integration of multifunctional pathways have prospected for the successful axon regeneration, and there are still many challenges for reconstructing a fully functional neural circuit. It is foreseeable that the key determinants will be identified to trigger central axon regeneration and functional recovery and mimics the preconditioning injury effect that has been applied clinically. Based on that understanding, novel pharmacological therapies recapitulating the preconditioning injury effect may be developed.
